# Preventive effects of minocycline in a neurodevelopmental two-hit model with relevance to schizophrenia

**DOI:** 10.1038/tp.2016.38

**Published:** 2016-04-05

**Authors:** S Giovanoli, H Engler, A Engler, J Richetto, J Feldon, M A Riva, M Schedlowski, U Meyer

**Affiliations:** 1 Physiology and Behavior Laboratory, ETH Zurich, Zurich, Switzerland; 2 Laboratory of Behavioral Neurobiology, ETH Zurich, Zurich, Switzerland; 3 Institute of Medical Psychology and Behavioral Immunobiology, University Hospital Essen, University of Duisburg-Essen, Essen, Germany; 4 Department of Pharmacological and Biomolecular Sciences, Università degli Studi di Milano, Milan, Italy; 5 Center of Excellence on Neurodegenerative Diseases, Department of Pharmacological and Biomolecular Sciences, Università degli Studi di Milano, Milan, Italy; 6 Institute of Pharmacology and Toxicology, University of Zurich-Vetsuisse, Zurich, Switzerland

## Abstract

Maternal immune activation can increase the vulnerability of the offspring to develop neuroimmune and behavioral abnormalities in response to stress in puberty. In offspring of immune-challenged mothers, stress-induced inflammatory processes precede the adult onset of multiple behavioral dysfunctions. Here, we explored whether an early anti-inflammatory intervention during peripubertal stress exposure might prevent the subsequent emergence of adult behavioral pathology. We used an environmental two-hit model in mice, in which prenatal maternal administration of the viral mimetic poly(I:C) served as the first hit, and exposure to sub-chronic unpredictable stress during peripubertal maturation as the second hit. Using this model, we examined the effectiveness of the tetracycline antibiotic minocycline (MINO) given during stress exposure to block stress-induced inflammatory responses and to prevent subsequent behavioral abnormalities. We found that combined exposure to prenatal immune activation and peripubertal stress caused significant deficits in prepulse inhibition and increased sensitivity to the psychotomimetic drugs amphetamine and dizocilpine in adulthood. MINO treatment during stress exposure prevented the emergence of these behavioral dysfunctions. In addition, the pharmacological intervention blocked hippocampal and prefrontal microglia activation and interleukin-1β expression in offspring exposed to prenatal infection and peripubertal stress. Together, these findings demonstrate that presymptomatic MINO treatment can prevent the subsequent emergence of multiple behavioral abnormalities relevant to human neuropsychiatric disorders with onset in early adulthood, including schizophrenia. Our epidemiologically informed two-hit model may thus encourage attempts to explore the use of anti-inflammatory agents in the early course of brain disorders that are characterized by signs of central nervous system inflammation during development.

## Introduction

Converging evidence implicates a role of immune mechanisms in normal and pathological brain development.^1, 2^ The antenatal period is highly sensitive to the damaging effects induced by environmental insults, and therefore, considerable efforts have been made to explore the impact of immune-mediated adversities such as prenatal infection in neuropsychiatric and neurological disorders with developmental components.^3^ Besides schizophrenia,^4^ maternal exposure to infection and/or inflammatory processes is associated with increased risk of bipolar disorder,^5^ autism,^6^ mental retardation^7^ and cerebral palsy.^8^ Prenatal infection and/or inflammation may thus represent a general vulnerability factor for neurodevelopmental brain disorders, so that the specificity of subsequent neuropathology and psychopathological symptoms is likely to be influenced by the genetic and environmental context in which the prenatal adversities occur.^9, 10^

Exposure to psychological trauma during sensitive periods of postnatal maturation is another environmental factor implicated in the etiology of major psychotic and affective disorders.^11, 12^ Using a translational mouse model, we have recently shown that combined exposure to prenatal immune challenge and peripubertal stress induces synergistic pathological effects on adult behavioral functions and neurochemistry.^13^ Hence, prenatal immune adversities can function as a neurodevelopmental disease primer that increases the offspring's vulnerability to the detrimental neuropathological effects of subsequent stress exposure during peripubertal life. In this environmental two-hit model, offspring exposed to combined prenatal immune challenge and peripubertal stress also showed signs of central nervous system inflammation in the form of microglia overactivation and hypersecretion of inflammatory cytokines in stress-sensitive brain areas.^13^ Intriguingly, these inflammatory abnormalities preceded the emergence of behavioral dysfunctions, the latter of which showed a delayed onset in adulthood.^13^ In view of these findings, we have hypothesized that the induction of peripubertal inflammation in prenatally primed offspring may interfere with the processes of neuronal maturation, thereby contributing to the delayed emergence of behavioral dysfunctions in adulthood.^13^ These putative processes may also have implications for preventive interventions. Indeed, the attenuation of inflammatory reactions in the event of peripubertal stress exposure may readily prevent the adult onset of behavioral pathologies.

In the present study, we tested this hypothesis by examining whether minocycline (MINO) administration during peripubertal stress exposure could block stress-induced inflammatory responses and prevent subsequent behavioral dysfunctions in offspring exposed to initial prenatal immune challenge. MINO is a broad-spectrum antibiotic of the tetracycline family that is believed to act through inhibition of secreted matrix metalloproteinase-9.^14, 15^ Importantly, MINO was found to be neuroprotective and highly effective in inhibiting microglia activation and associated neuroinflammation in numerous pathological conditions,^16, 17, 18^ including models of intense prenatal or neonatal immune activation with relevance to schizophrenia.^19, 20, 21, 22^ Another important property of MINO is that it readily crosses the blood–brain barrier^15^ and can be easily administered via regular drinking water, eliminating potential confounds associated with stress effects induced by more invasive routes of administration. Therefore, we opted for MINO as a suitable pharmacological intervention to test our hypothesis that reducing stress-induced inflammatory reactions in prenatally primed offspring may prevent the subsequent emergence of behavioral dysfunctions in adulthood.

We conducted these investigations using the recently established mouse model of combined prenatal immune activation and peripubertal stress in mice.^13^ In this model, prenatal immune activation is induced by the viral mimetic polyriboinosinic–polyribocytidilic acid (poly(I:C)), a synthetic analog of double-stranded RNA that induces a cytokine-associated viral-like acute phase response.^23^ Offspring born to poly(I:C)-exposed or control mothers are then left undisturbed or exposed to variable and unpredictable stress during peripubertal development.^13^ Using this environmental two-hit model, the present study tested whether MINO treatment during the course of peripubertal stress exposure would prevent the adult emergence of behavioral abnormalities identified previously,^13^ including increased anxiety-like behavior, impaired sensorimotor gating and potentiated sensitivity to psychotomimetic drugs. These behavioral investigations were conducted after a MINO washout period of 4 weeks. Hence, our preventive approach is contrary to the recent investigations assessing behavioral effects of MINO in developmental immune activation models, where the drug was given either throughout behavioral testing,^19^ or where behavioral examinations started 1 day after the last MINO exposure.^20, 21^ Even though the findings from these latter models suggest that MINO is capable of correcting infection-induced behavioral abnormalities relevant to schizophrenia and related disorders, these effects arise from symptomatic rather than preventive interventions. The present study is thus expected to provide novel information with regard to the preventive potential of presymptomatic MINO treatment in a multi-factorial model of schizophrenia and related disorders.

## Materials and methods

### Animals

C57BL6/J mice (Jackson Laboratory; distributed via Charles River Laboratories, Sulzfeld, Germany) were used throughout the study. A description of the animal housing and maintenance is provided in the Supplementary Information. The number of animals used per group is summarized in Supplementary Tables 1 and 2). All procedures described in the present study were approved by the Cantonal Veterinarian's Office of Zurich, Switzerland. All efforts were made to minimize the number of animals used and their suffering.

### Prenatal immune activation

Pregnant dams on gestation day 9 (GD9) received either a single injection of poly(I:C) (potassium salt; Sigma-Aldrich, Buchs, Switzerland) at a dose of 1 mg kg^−1^ or vehicle (sterile pyrogen-free 0.9% NaCl) as fully described the Supplementary Information. The dose of poly(I:C) was chosen based on our previous findings showing that this immunological manipulation leads to modest and transient cytokine elevations in the maternal host.^13^ Here, we confirmed the transient induction of one key inflammatory cytokine, namely interleukin (IL)-6, in the maternal host following treatment with the chosen dose of poly(I:C) (Supplementary Figure 1). The gestational stage (that is, GD9) roughly corresponds to the first trimester of human pregnancy with respect to fetal developmental biology and was chosen based on our previous findings.^13^

### Peripubertal stress exposure

Male and female offspring born to poly(I:C)-treated (POL) or saline-treated control (CON) mothers were weaned on postnatal day (PND) 21 and caged as littermates of two to three animals per cage. Litters with four or more animals per sex were split into separate cages and were assigned to different treatments, to minimize potential confounds associated with litter effects. At peripubertal age, between PND 30 and 40, mice were then either exposed to variable and unpredictable stress (S+) or left undisturbed (=no stressor; S−). According to protocols established before,^13^ the stress procedure included exposure to five distinct stressors ((1) electric foot shock; (2) restraint stress; (3) swimming stress; (4) food deprivation; (5) repeated home cage changes) applied on alternate days. A detailed description of the peripubertal stress protocol is given in the Supplementary Information. All animals of a particular housing cage underwent the same peripubertal procedures in terms of stress exposure and drug treatment (see below). The allocation of cages to the peripubertal procedures was randomized.

The peripubertal period was selected based on our previous findings showing that prenatally immune-challenged animals exhibit a maximal sensitivity for stress-induced neuropathological changes at this age.^13^ Besides the drastic hormonal changes, this developmental period is also characterized by considerable neuroplastic rearrangements during which neural circuits mature and thereby undergo various structural and functional changes.^24, 25, 26^ Environmental insults during this phase of postnatal maturation are therefore likely to interfere with normal brain development and thus represent a relevant vulnerability factor in the development of psychiatric diseases.^13, 26^

### MINO treatment

MINO hydrochloride (MINO; Sigma-Aldrich) was dissolved in regular tap water and provided via regular drinking bottles to avoid additional peripubertal stress exposure resulting from daily injections. Vehicle (VEH)-exposed animals received tap water only. MINO was administered during the course of peripubertal stress exposure to block the central inflammatory responses to stress.^13^ More specifically, MINO treatment started 24 h before exposure to the first stressor on PND 30 and ended after completion of the stress procedure on PND 40. It was administered at a dose of 30 mg kg^−1^ per day (per os in drinking water) based on our pilot studies showing that MINO at this dose is highly effective in preventing the up- and downregulation of pro-inflammatory cytokine expression and neuron–microglia inhibitory signaling typically emerging in the brains of prenatally immune-challenged offspring exposed to acute pubertal stress (see Supplementary Figure 2). The dosage used here is in the range of other oral MINO regimens known to block stress-induced microglia activation and neuronal maladaptations.^17^ For each cage, the dose of MINO was calculated based on the average liquid consumption and body weight per cage; this was adjusted every second day based on the liquid consumption and body weight assessed on the preceding 2 days.

### Behavioral analyses

Behavioral tests in MINO- or VEH-treated offspring subjected to single or combined immune activation and stress started 4 weeks after exposure to the last stressor, that is, between PND 70 and PND 90. This testing age was selected because it corresponds to the early-adult stage of maturation when the combined effects of prenatal immune activation and peripubertal stress become behaviorally manifested.^13^ In a first cohort of animals, MINO or VEH was given to CON and POL offspring with (S+) or without (S−) peripubertal stress exposure. The inclusion of the latter condition served to ascertain whether the MINO administration regimen might be associated with unwanted side effects on adult behavioral functions in non-stressed animals. Animals from the first cohort were tested in the elevated plus maze test to measure innate anxiety-like behavior, and in the test of prepulse inhibition (PPI) of the acoustic startle reflex to assess sensorimotor gating. The sample size consisted of 12–18 animals per group based on our previous findings.^13^ A second cohort of animals (*N*=12–16 animals per group) was used to explore the beneficial effects of MINO against hypersensitivity to the psychotomimetic drugs amphetamine (AMPH) and dizocilpine (MK-801). For this purpose, we only included stressed (S+) POL and CON offspring (with or without MINO treatment) because the preceding tests using the first cohort of animals did not reveal any significant behavioral effects of MINO in non-stressed (S−) animals. The tests assessing AMPH and MK-801 sensitivity were evaluated in terms of drug-induced changes in locomotor activity and were conducted in two independent sub-groups of animals to avoid repeated exposure to psychotomimetic drugs. A detailed description of the behavioral test apparatuses and procedure is provided in the Supplementary Information.

### Immunohistochemical analyses

To ascertain the effects of MINO on microglia activation, we performed immunohistochemical analyses of ionized calcium-binding adaptor molecule 1 (Iba1) and cluster of differentiation 68 (CD68), two cellular markers expressed by the entire (non-activated and activated) and primarily activated microglia population, respectively.^27^ In addition, we explored the effects of MINO treatment on pro-inflammatory cytokine expression by immunohistochemical evaluations of IL-1β protein to confirm our gene expression pilot data (see Supplementary Figure 2). For all immunohistochemical evaluations, MINO or VEH were given to CON and POL offspring during the course of the peripubertal stress exposure as described above, and the animals were then killed 24 h after exposure to the last stressor on PND 41. Only one offspring per litter was allocated for peripubertal euthanasia to minimize potential confounds associated with litter effects. The age of euthanasia was chosen based on our previous findings, showing that the inflammatory effects of combined exposure to the two environmental insults are transient and are primarily manifested in the event of and/or shortly after experience of the second environmental hit in puberty.^13^ A detailed description of the methods used for the immunohistochemical analyses is provided in the Supplementary Information.

Iba1-, CD68- and IL-1β-positive cells were counted in the hippocampus and prefrontal cortex using unbiased stereological estimations as fully described in the Supplementary Information. In addition to the stereological estimates, we characterized microglia morphology by quantifying the cell soma area and number of primary processes of Iba1-positive microglia cells. The methods used for the assessment of microglia morphology are also fully described in the Supplementary Information. All quantifications were performed in the hippocampus and the prefrontal cortex because these brain regions were shown to be highly sensitive to stress-induced inflammatory changes.^13^ For all stereological and morphological analyses, the experimenter was blind to the animals' treatment conditions and only had access to codes in the form of a number.

### Statistical analyses

All data met the assumptions of normal distribution and equality of variance and were analyzed using analysis of variance (ANOVA) to identify the main effects of sex, prenatal immune treatment, postnatal stress treatment and preventive MINO treatment, as well as their interactions. The individual ANOVAs used for each test are outlined in Supplementary Tables 3–5, which also summarize the statistical outcomes obtained by ANOVA. Fisher's least significant difference *post hoc* tests were used whenever significant interactions were obtained by the initial ANOVAs. No pre-established inclusion/exclusion criteria were used. Statistical significance was set at *P*<0.05. All statistical analyses were performed using the statistical software StatView (version 5.0; Abacus, Phoenix, AZ, USA) implemented on a PC running the Windows XP operating system.

## Results

### MINO fails to prevent the emergence of increased anxiety-like behavior induced by peripubertal stress

**Figure 1:**
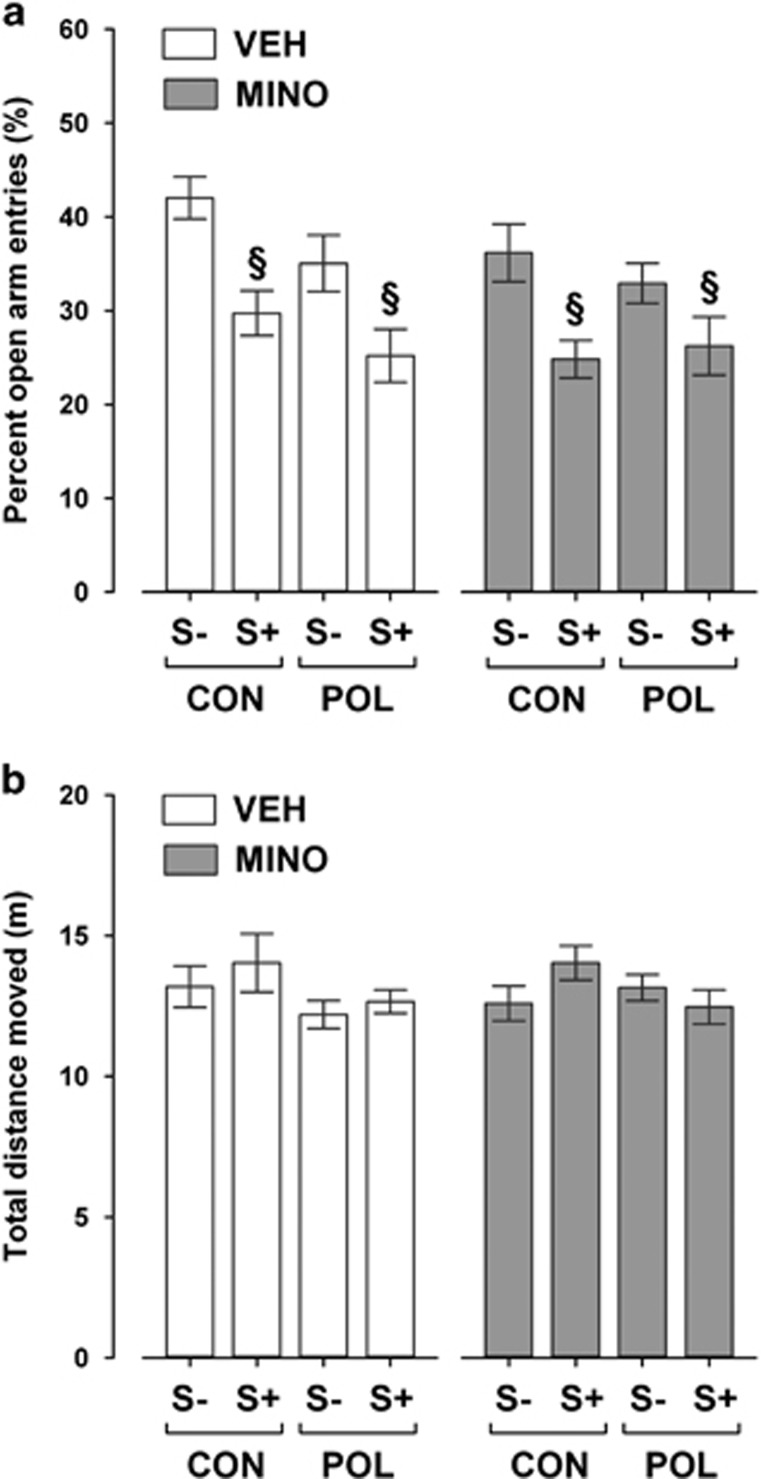
Elevated plus maze performance in adult offspring exposed to single or combined prenatal immune activation and peripubertal stress with or without preventive minocycline (MINO) treatment. Pregnant mice were injected with 1 mg kg^−1^ poly(I:C) (POL) or physiological saline (control (CON)), and the resulting offspring were subjected to sub-chronic stress (S+) or left undisturbed (S−) during peripubertal maturation. During the stress procedure, half of the animals received MINO treatment (30 mg kg^−1^ per day, per os in drinking water), and the other half vehicle (VEH; = regular tap water) treatment. (**a**) The bar plot depicts percent open arm entries (%). ^§^*P*<0.001, reflecting the significant main effect of peripubertal stress. *N*=12–18 per group. (**b**) The bar plot shows the total distance moved (m) during the entire test period. *N*=12–18 per group. All data are means±s.e.m.

First, we explored whether the MINO treatment would prevent the emergence of increased anxiety-like behavior typically seen in offspring exposed to peripubertal stress with or without additional prenatal immune challenge.^13^ Consistent with our previous findings,^13^ we found that peripubertal stress increased anxiety-like behavior in the elevated plus maze test regardless of the prenatal immune histories. Hence, stressed offspring displayed a significant reduction in the frequency to enter the open arms compared with non-stressed animals, and this effect similarly emerged in the two prenatal conditions (see Figure 1a). The stress-induced changes in open arm frequencies were not accompanied by concomitant alterations in basal locomotor activity as indexed by the total distance moved on the elevated plus maze (Figure 1b). MINO treatment failed to prevent the stress-induced changes in anxiety-like behavior: a comparable reduction in open arm frequencies was observed in stressed animals regardless of whether they received MINO or VEH (Figure 1a). MINO also did not affect the animals' basal locomotor activity scores as measured by the distance moved during the elevated plus maze test (Figure 1b).

### MINO prevents the development of sensorimotor gating deficiency following combined prenatal immune activation and peripubertal stress

**Figure 2:**
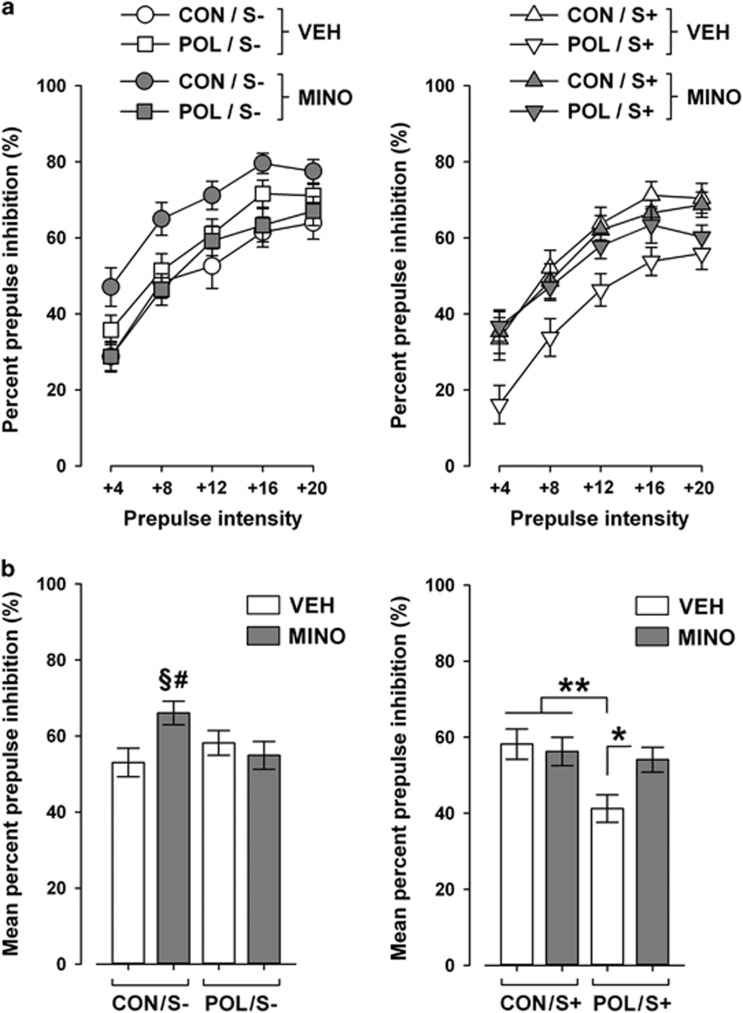
Prepulse inhibition of the acoustic startle reflex in adult offspring exposed to single or combined prenatal immune activation and peripubertal stress with or without preventive minocycline (MINO) treatment. Pregnant mice were injected with 1 mg kg^−1^ poly(I:C) (POL) or physiological saline (control (CON)), and the resulting offspring were subjected to sub-chronic stress (S+) or left undisturbed (S−) during peripubertal maturation. During the stress procedure, half of the animals received MINO treatment (30 mg kg^−1^ per day, per os in drinking water), and the other half vehicle (VEH; = regular tap water) treatment. (**a**) The line plots depict percent prepulse inhibition as a function of increasing prepulse intensities (dB above background of 65 dB). (**b**) The bar plots show the mean percent prepulse inhibition across all five prepulse intensities. ^§^*P*<0.01, reflecting the significant difference between CON/S−/MINO offspring and CON/S−/VEH offspring; ^#^*P*<0.05, reflecting the significant difference between CON/S−/MINO offspring and POL/S−/MINO offspring; *P<0.05 and **P<0.01, reflecting the indicated differences in the S+ groups. *N*=12–18 per group. All data are means±s.e.m.

In a next series of investigations, we tested whether MINO treatment might be effective in preventing the adult emergence of sensorimotor gating deficiency following combined prenatal immune activation and peripubertal stress.^13^ Sensorimotor gating was evaluated using the paradigm of PPI of the acoustic startle reflex. In line with our previous findings,^13^ we revealed interactive effects between the two environmental manipulations on PPI disruption in adulthood: Neither prenatal immune activation alone nor stress alone was sufficient to significantly affect PPI in the VEH condition, but only the combination of the two insults resulted in a significant attenuation of PPI (Figure 2). Peripubertal MINO administration prevented the disruption of PPI in offspring exposed to combined immune activation and stress (Figure 2). Indeed, MINO treatment in stressed POL offspring significantly elevated PPI scores to levels found in VEH-treated CON offspring with or without additional stress exposure (Figure 2). MINO administration also increased PPI levels in non-stressed offspring. This effect, however, was only evident in non-stressed CON animals, likely because of their relatively low basal PPI scores (Figure 2).

Single or combined exposure to immune activation and stress did not affect the reactivity to pulse-alone trials or prepulse alone trials (Supplementary Table 6). Peripubertal MINO treatment also did not influence these dependent variables, suggesting that the beneficial effects of the drug on PPI scores in stressed POL offspring are independent of possible influences on startle reactivity and prepulse-induced reactivity.

### MINO prevents the emergence of hypersensitivity to psychotomimetic drugs induced by combined prenatal immune activation and peripubertal stress

**Figure 3:**
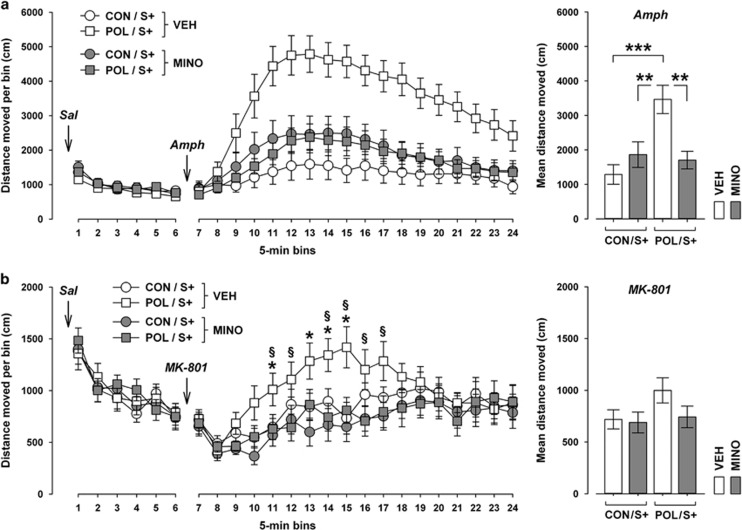
Effects of minocycline (MINO) on the locomotor responses to acute challenge with psychotomimetic drugs in adult offspring exposed to prenatal control treatment or immune activation with additional stress exposure in puberty. Pregnant mice were injected with 1 mg kg^−1^ poly(I:C) (POL) or physiological saline (control (CON)), and the resulting offspring were subjected to sub-chronic stress (S+) during peripubertal development. During the stress procedure, half of the animals received MINO treatment (30 mg kg^−1^ per day, per os in drinking water), and the other half vehicle (VEH; = regular tap water) treatment. (**a**) Locomotor reaction to the indirect dopamine receptor agonist amphetamine (Amph; 2.5 mg kg^−1^, i.p.). The line plot depicts the distance moved in an open field arena to initial vehicle (saline (Sal)) treatment and subsequent Amph treatment as a function of successive 5-min bins. The bar plot depicts the mean distance moved after Amph treatment. **P<0.01 and ***P<0.001; *N*=12–16 per group. (**b**) Locomotor reaction to the non-competitive NMDA receptor antagonist dizocilpine (MK-801; 0.15 mg kg^−1^, i.p.). The line plot shows the distance moved in an open field arena to initial vehicle (Sal) treatment and subsequent MK-801 treatment as a function of successive 5-min bins. *P<0.05, reflecting the significant difference between VEH-exposed CON/S+ and POL/S+ offspring at individual bins; ^§^*P*<0.05, reflecting the significant difference between VEH-exposed POL/S+ and MINO-treated POL/S+ offspring at individual bins. *N*=16 per group. All data are means±s.e.m.

Another pathological feature emerging following combined exposure to prenatal immune activation and peripubertal stress is the adult onset of increased sensitivity to psychotomimetic drugs.^13^ We have previously revealed interactive effects between these two environmental manipulations on the development of potentiated locomotor reactions to the indirect dopamine receptor agonist AMPH and the non-competitive NMDA receptor antagonist MK-801.^13^ Here, we tested whether MINO treatment may be effective in preventing these abnormalities. Consistent with our previous report,^13^ AMPH-induced locomotor activity in the open field test was significantly increased in POL offspring exposed to peripubertal stress compared with stressed CON offspring (see Figure 3a). MINO treatment fully prevented this pathological phenotype in stressed POL offspring without significantly influencing AMPH-induced activity in stressed CON offspring.

MINO treatment also prevented the potentiation of MK-801-induced hyperactivity induced by combined prenatal immune activation and peripubertal stress (see Figure 3b). Similar to its effects against AMPH hyperactivity, MINO treatment reduced the MK-801-induced hyperlocomotor responses in stressed POL offspring to levels found in stressed CON offspring that had been treated with VEH or MINO.

MINO treatment did not affect basal locomotor activity as assessed during the initial saline exposure phase that preceded the subsequent AMPH (Figure 3a) or MK-801 (Figure 3b) exposure phase. These effects are consistent with the outcomes in the elevated plus maze test (Figure 1b) and suggest that the beneficial effects of MINO against psychotomimetic drugs-induced hyperactivity emerge independently of possible influences on basal locomotor activity.

### MINO blocks microglia activation and IL-1β expression in offspring with combined exposure to prenatal immune activation and stress in puberty

We have previously demonstrated that offspring born to immune-challenged mothers show an increased sensitivity to stress-induced activation of hippocampal and prefrontal microglia cells.^13^ Intriguingly, such microglia abnormalities in prenatally immune-challenged offspring were clearly evident shortly after exposure to the last stressor in peripubertal life, but absent when the offspring reached adulthood.^13^ Hence, prenatal immune activation can prime latent microglia overactivation in response to stress, which precedes the adult onset of multiple behavioral abnormalities. Here, we ascertained the effectiveness of MINO to block the activation of microglia cells in offspring with initial prenatal immune activation exposed to stress relative to prenatal CON offspring exposed to stress.

**Figure 4:**
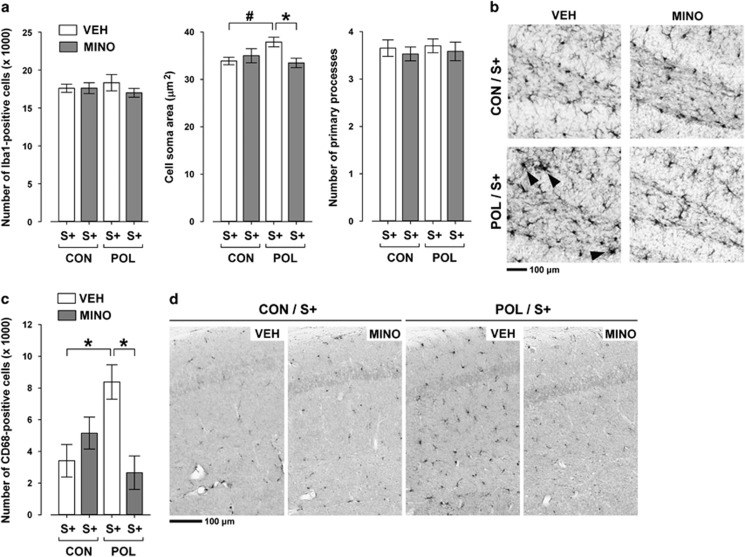
Effects of minocycline (MINO) on microglia abnormalities in the hippocampus of stressed offspring born to control or gestationally immune-challenged mothers. Pregnant mice were injected with 1 mg kg^−1^ poly(I:C) (POL) or physiological saline (control (CON)), and the resulting offspring were subjected to sub-chronic stress (S+) during peripubertal maturation. During the stress procedure, half of the animals received MINO treatment (30 mg kg^−1^ per day, per os in drinking water), and the other half vehicle (VEH; = regular tap water) treatment. (**a**) The bar plots depict the stereological estimates of Iba1-positive cells, as well as cell soma area and number of primary processes of Iba1-positive microglia. *P<0.05 and ^#^*P*=0.07; *N*=5 per group. (**b**) The photomicrographs show representative sections stained with anti-Iba1 antibody. Note the enlargement of the cell soma area in Iba1-positive microglia cells in VEH-treated POL/S+ offspring relative to the other groups (indicated by the black arrow head). (**c**) The bar plot shows the stereological estimates of activated CD68-positive microglia cells. *P<0.05, *N*=5 per group. (**d**) The photomicrographs show representative coronal brain sections stained with anti-CD68 antibody. Note the increase in microglial CD68 expression in VEH-treated POL/S+ offspring relative to the other groups. All data are means±s.e.m.

MINO treatment did not change the number of Iba1-positive cells in the hippocampus (Figure 4a) and prefrontal cortex (Supplementary Figure 3), suggesting that it did not influence the overall density of the entire (non-activated and activated) microglia cell population. On the other hand, the pharmacological intervention was effective in preventing morphological and cellular signs of microglia activation in prenatally primed offspring: MINO normalized the enlargement of Iba1-positive cell soma areas in the hippocampus of stressed POL offspring to levels present in stressed CON offspring (Figure 4b), and it fully blocked the induction of hippocampal (Figures 4c and d) and prefrontal (Supplementary Figure 3) CD68 expression typically seen in offspring with combined exposure to prenatal immune activation and peripubertal stress.

**Figure 5:**
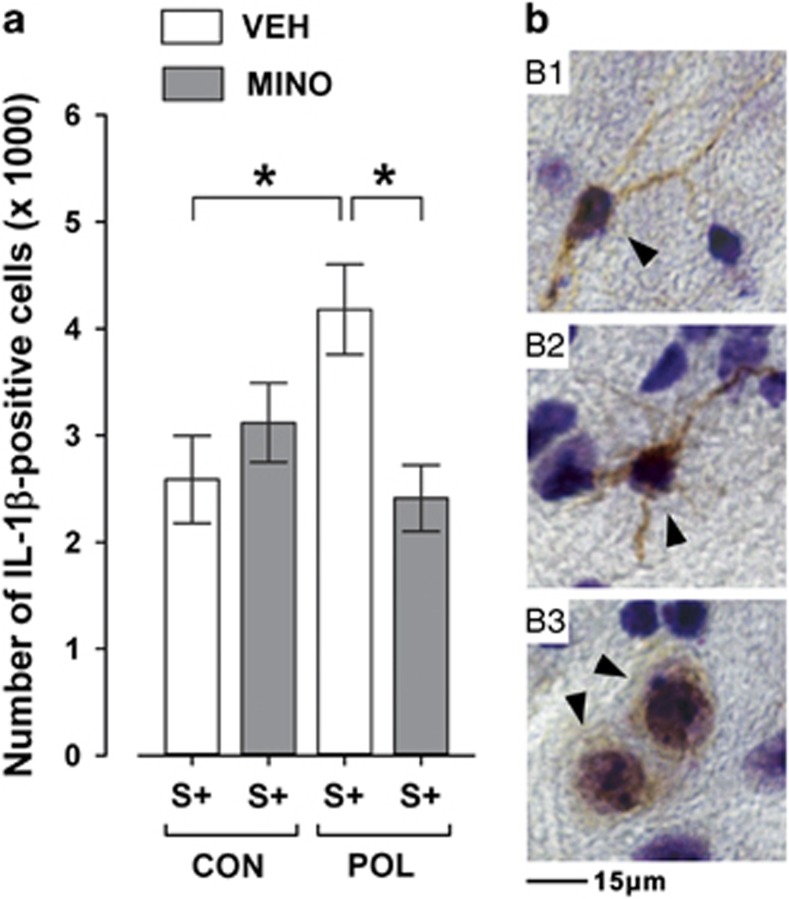
Effects of minocycline (MINO) on IL-1β-positive cells in the hippocampus of stressed offspring born to control or gestationally immune-challenged mothers. Pregnant mice were injected with 1 mg kg^−1^ poly(I:C) (POL) or physiological saline (control (CON)), and the resulting offspring were subjected to sub-chronic stress (S+) during peripubertal maturation. During the stress procedure, half of the animals received MINO treatment (30 mg kg^−1^ per day, per os in drinking water), and the other half vehicle (VEH; = regular tap water) treatment. (**a**) The bar plot shows the stereological estimates of IL-1β-positive cells. *P<0.05, *N*=5 per group. (**b**) The photomicrographs (b1–b3) show representative IL-1β immunoreactivity (indicated by the black arrowheads) in the hippocampal formation visualized with a Nissl/IL-1β double-staining. Note that IL-1β immunoreactivity was present in cells displaying noticeable immunoreactive processes (b1 and b2) and in cells with no immunoreactive processes (b3). All data are means±s.e.m.

Consistent with our previous investigations^13^ and pilot data (Supplementary Figure 2), we found that POL offspring exhibited significantly enhanced IL-1β expression in response to peripubertal stress compared with stress exposure in CON offspring (Figure 5). MINO administration prevented this augmentation in inflammatory cytokine expression, so that the numbers of IL-1β-positive cells were highly comparable between VEH-treated CON offspring with stress and MINO-treated POL offspring with stress (Figure 5).

## Discussion

Our study demonstrates that administration of the tetracycline antibiotic MINO during the course of peripubertal stress exposure prevents the subsequent emergence of behavioral abnormalities in offspring with a history of prenatal immune activation. In our environmental two-hit model, combined exposure to prenatal immune challenge and peripubertal stress induced synergistic pathological effects on adult behavioral functions relevant primarily to schizophrenia,^13^ which included impairments in PPI and increased sensitivity to the psychotomimetic drugs AMPH and MK-801. Peripubertal MINO administration proved to be highly efficient in blocking the development of these abnormalities. Our results further suggest that these preventive effects of MINO are largely sex-independent, as we did not reveal any significant four-way interactions between prenatal immune activation, postnatal stress exposure, preventive MINO treatment and sex. This is consistent with the findings by Zhu *et al.*^22^ who reported no sex-dependent effects of MINO in the correction of behavioral deficits and microglial activation following prenatal exposure to high doses of poly(I:C) in mice. Thus, hormonal differences between male and female offspring during peripubertal maturation seem to have a minor influence on the effectiveness of the MINO treatment in preventing long-term behavioral pathologies.

In contrast to its effects on sensorimotor gating and psychosomatic drug sensitivity, however, MINO failed to prevent the stress-induced increase in anxiety-like behavior. The latter developed after peripubertal stress regardless of whether the offspring had been exposed to the first environmental hit in prenatal life. These findings indicate that MINO is not simply associated with general protective effects against stress-induced behavioral abnormalities, but rather, it seems to be particularly efficient in preventing pathological effects that require the combination of two environmental adversities, in this case prenatal immune activation followed by peripubertal stress exposure.

The beneficial effects of MINO on PPI impairments and hypersensitivity to psychotomimetic drugs are consistent with the drug's beneficial effects revealed in other rodent models that capture abnormalities in these behavioral domains.^28, 29^ Notably, our data corroborate the recent findings obtained in more severe maternal or neonatal immune activation models, in which exposure to high doses of poly(I:C) or the bacterial lipopolysaccharide in rats or mice resulted in persistent microglia activation, increased inflammatory cytokine production and PPI impairments even in the absence of additional environmental adversities such as stress.^19, 20, 21, 22^ Consistent with our results, chronic MINO treatment effectively normalized these inflammatory abnormalities and restored the disruption of behavioral dysfunctions in these models of intense early-life immune challenge.^19, 20, 21, 22^ Our study provides two important extensions to these recent findings. First, our data are based on a multi-factorial model of schizophrenia and related disorders that incorporates two etiologically relevant (environmental) risk factors rather than one only. Second, we conducted all behavioral investigations after a comparatively long drug washout period of 4 weeks, whereas behavioral examinations in previous developmental immune activation models took place either shortly after, or concurrently with, MINO treatment.^20, 21^ Hence, previous developmental immune activation models reported symptomatic effects of MINO treatment against schizophrenia-related abnormalities, while our study probed preventive effects of the drug.

There are also important distinctions between the present two-hit model and more severe early-life immune activation models with respect to the persistence of neuroimmune changes. Maternal or neonatal exposure to intense immune challenges stimuli can cause long-lasting inflammatory changes in the offspring's brains, some of which can persist from juvenile to adult stages of life.^19, 20, 21, 22, 30, 31^ These enduring changes contrast the nature of inflammatory signs in our two-hit model, in which increased microglia activation and brain inflammatory cytokine production emerge in immune-challenged offspring only if they are exposed to additional environmental adversities such as peripubertal stress. Hence, a second environmental hit is required to unmask latent neuroimmune pathologies following priming by mild prenatal immune activation.^13^ On unmasking, the neuroimmune anomalies are transient and are evident only during the course and shortly after exposure to the second environmental hit in puberty.^13^ Against these backgrounds, our findings highlight that MINO can exert beneficial effects even if its administration is restricted to a developmental period of transient brain inflammation that precedes the adult onset of behavioral abnormalities. These data may thus encourage clinical attempts to explore the preventive potential of MINO when administered during early or even presymptomatic phases of chronic mental illnesses with delayed onset in early adulthood, including schizophrenia and related psychotic disorders.^32^ Thus far, MINO has demonstrated some positive effects in the treatment of neuropsychiatric disorders once overt psychopathological symptoms manifest,^33, 34, 35^ but its preventive potential when given during earlier (presymptomatic or first onset) phases still awaits examination. Such early anti-inflammatory interventions may indeed be effective in view of the converging findings, suggesting that altered inflammatory processes are also relevant before and/or during the onset of full-blown neuropsychiatric disease.^36, 37, 38, 39, 40^ However, presymptomatic treatments also raise important issues regarding unwanted side effects as not all persons at risk transition to disease. We found that presymptomatic MINO administration did not lead to any adverse behavioral outcome in control animals that were exposed to only one or none of the environmental insults. At the same time, however, the early intervention was effective in preventing the development of functional abnormalities in offspring exposed to combined immune activation and stress. Even though these findings are highly consistent with other studies,^19, 20, 21^ the limited set of behavioral tests performed here cannot fully exclude potential side effects of the MINO treatment in control (or healthy) subjects that would otherwise not go on to develop adult behavioral dysfunctions. This aspect is, however, of high importance and clearly warrants further attention in future studies.

The mechanisms through which MINO prevented the subsequent emergence of behavioral abnormalities remain unknown and require further investigation. It can be excluded, however, that the drug's beneficial effects are related to a direct action on neurobehavioral processes in adulthood. The main reason for this assumption is that MINO was administered only during a restricted period in peripubertal life, which was followed by a drug washout period of 4 weeks. It is therefore more likely that MINO may have exerted its beneficial effects by mitigating abnormalities in brain maturation, which are primed by (mild) prenatal immune activation and set in motion by subsequent exposure to peripubertal stress. The peripubertal period is arguably a very sensitive and dynamic period of neuronal and hormonal rearrangements that prepare the growing organism to the demands in adult life,^41, 42^ so that altered neuroinflammatory processes occurring during this period can be expected to have long-lasting consequences on adult brain functions.^43^

Based on the present findings, it would be tempting to speculate that abnormal microglia activation during peripubertal brain maturation may assume a key role in these processes. Microglia play crucial roles in both neuronal protection and pathology, and are often referred to as a ‘double-edged sword'.^44, 45^ On the one hand, they secrete neurotrophic factors pivotal for cellular repair, and recruit immune cells into the brain for clearance of infection or cellular debris. On the other hand, chronic or exaggerated microglia activation is linked to excessive secretion of pro-inflammatory factors and has been linked to neurodegenerative processes.^44, 45^ Of particular interest for the present findings may be the recently described role of microglia in patterning and wiring of the developing and maturing brain, whereby altered microglia functions can negatively influence various processes such as programmed cell death, activity-dependent synaptic pruning and synapse maturation.^46, 47^ At the present stage, however, the hypothesized mechanistic role of abnormal microglia activation still needs to be met with caution in the present two-hit model. Indeed, we did not establish a direct link between peripubertal inflammation and disruption of neuronal substrates across postnatal maturation, nor did we examine alternative mechanism by which MINO could exert beneficial effects in this two-hit model. Despite the converging evidence that MINO can effectively block microglia activation and subsequent inflammatory processes,^16, 17, 18^ it is arguably not a specific microglia inhibitor, but instead, it is associated with various other pharmacological properties.^14, 15, 48^ Nonetheless, our findings suggest that MINO exerts beneficial effects in a developmental disruption model with inflammatory components that bears etiological relevance to brain disorders with delayed onsets in adulthood.^13^

In conclusion, our findings provide novel evidence showing that the tetracycline antibiotic MINO exhibits preventive effects against adult behavioral abnormalities in a developmental two-hit model relevant to schizophrenia and related disorders. Our preclinical data may encourage attempts to explore the use of transient MINO treatment for preventive reasons, especially for brain disorders that are characterized by a delayed onset in adulthood.
