# Supplementary material for: Preventive effects of minocycline in a neurodevelopmental two-hit model with relevance to schizophrenia
Source: Transl Psychiatry. 2016 Apr 5;6(4):e772–. doi: 10.1038/tp.2016.38 (PMC4872396; doi:10.1038/tp.2016.38)
Supplement: Supplementary Information [file tp201638x1.doc]

**SUPPLEMENTARY INFORMATION**

**Preventive effects of minocycline in a neurodevelopmental two-hit model with relevance to schizophrenia**

Sandra Giovanoli^1,2,§^, Harald Engler^3,§^, Andrea Engler^3^, Juliet Richetto^4,5,6^, Joram Feldon^2^, Marco A. Riva^4,5^, Manfred Schedlowski^3^, Urs Meyer^1,2,6,*^

^1^Physiology and Behavior Laboratory, ETH Zurich, Schwerzenbach, Switzerland.

^2^Laboratory of Behavioral Neurobiology, ETH Zurich, Schwerzenbach, Switzerland.

^3^Institute of Medical Psychology and Behavioral Immunobiology, University Hospital Essen, University of Duisburg-Essen, Essen, Germany.

^4^Department of Pharmacological and Biomolecular Sciences, Università degli Studi di Milano, Milan, Italy.

^5^Center of Excellence on Neurodegenerative Diseases, Department of Pharmacological and Biomolecular Sciences, Università degli Studi di Milano, Milan, Italy.

^6^Institute of Pharmacology and Toxicology, University of Zurich-Vetsuisse, Zurich, Switzerland.

^§^These authors contributed equally to the present study.

*Correspondence: Prof. Urs Meyer (PhD)

Institute of Pharmacology and Toxicology, University of Zurich-Vetsuisse

Winterthurerstrasse 260, 8057 Zurich, Switzerland

E-mail: urs.meyer@vetpharm.uzh.ch

Tel.: +41 44 635 88 44; Fax.: +41 44 635 89 10

**Supplementary Methods**

**Animals**

C57BL6/J female and male breeders (Jackson Laboratory) were obtained at the age of 10-12 weeks. Breeding began after 2 weeks of acclimatization to the animal holding rooms, which were temperature- and humidity-controlled (21±1 °C, 55±5 %) holding facilities under a reversed light-dark cycle (lights off: 8:00 A.M. to 8:00 P.M.). All animals had *ad libitum* access to standard rodent chow (Kliba 3430, Klibamuehlen, Kaiseraugst, Switzerland) and water unless specified otherwise. All procedures described in the present study had been previously approved by the Cantonal Veterinarian's Office of Zurich and are in agreement with the principles of laboratory animal care in the Guide for the Care and Use of Laboratory Animals (National Institutes of Health Publication No. 86-23, revised 1985). All efforts were made to minimize the number of animals used and their suffering.

**Prenatal immune activation**

For the purpose of the maternal immunological manipulation during pregnancy, female mice were subjected to a timed-mating procedure as fully described previously (1). Pregnant dams received either a single injection of poly(I:C) (potassium salt; Sigma-Aldrich) or vehicle on gestation day 9 (GD9). This gestational stage (i.e., GD9) was selected based on our previous findings (2). GD9 in mice corresponds approximately to the first trimester of human pregnancy with respect to developmental biology and percentage of gestation (3). Poly(I:C) (1 mg/kg) was dissolved in sterile pyrogen-free 0.9% NaCl (= vehicle) solution to yield a final concentration of 0.2 mg/ml and was administered via the intravenous (i.v.) route at the tail vein under mild physical constraint (see below). All solutions were freshly prepared at the day of administration and injected with a volume of 5 ml/kg. All animals were returned to their home cages immediately after the injection procedure.

For the purpose of the i.v. injections, each animal's tail was first bathed in 40°C water to better visualize and dilate the tail veins. Subsequently, the animals were mildly restrained using a nonrestrictive Plexiglas restrainer (561-RC, Plas Labs) and the substance was injected either into the right or left tail vein. Administration of the chosen dose of poly(I:C) resulted in an acute systemic inflammatory response in the maternal host as evident from the transient increase in interleukin (IL)-6, a key inflammatory cytokine (see **Supplementary Figure 1**).

**Peripubertal stress exposure**

The peripubertal stress procedure was performed according to protocols established before (2) and included 5 distinct stressors applied on alternate days between PND 30 and 40 in the following order: 1. Electric foot shock (day 1); 2. Restraint stress (day 3); 3. Food deprivation (day 5); 4. Forced swimming stress (day 7); 5. Multiple changes of home cage embedding (day 9). A one-day resting period followed each stressor day.

• *Exposure to electric foot shock (day 1).* Four identical freezing chambers (Coulbourn Instruments, Allentown, PA) were used, each installed in a sound insulated, wooden cabinet. Each cabinet was equipped with 16 stainless steel bars (diameter: 4 mm) spaced 10 mm apart from center to center, through which scrambled electric foot shocks could be delivered by a Coulbourn Precision Regulated Animal Shocker (Model E13-12). Animals were restricted to a semi-circular (19 cm in diameter; 30 cm height) Plexiglas enclosure, which was positioned over the grid. The lights were turned off throughout the entire period, and additional odour stimuli were presented by placing a compressed tissue saturated with SteriliumTM solution (Bode Chemie Hamburg, Germany) into the wooden cabinets. The chambers were cleaned after each run, and SteriliumTM tissues were saturated again. Each animal was first placed into the chamber for an initial period of 3 min, after which it received 3 mild electric foot shocks (0.25 mA), each lasting 1 sec. The electric foot shocks were separated by intervals of 3 min. The session ended with an additional 3 min period, in which no shocks were delivered.

• *Exposure to restraint stress (day 3).* Each animal assigned to the peripubertal stress exposure was kept in a transparent plastic tube (diameter: 3 cm; length: 11.5 cm) for 45 min. The restrainer tubes contained drilled holes (2 mm in diameter) so as to facilitate oxygen supply. The tubes were tapped on a wooden table which was placed in a brightly lit testing room. The animals were immediately returned to their home cages at the end of the restraint stress procedure.

• *Exposure to food deprivation (day 5).* Littermates assigned to peri-pubertal stress were subjected to 20-h food deprivation. For this purpose, animals were kept in their homes cages, and the food was removed at 12h00 on day 5 and added again the next day at 08h00. Animals had free access to water during the entire food deprivation period.

• *Exposure to forced swim stress (day 7).* The apparatus used for the forced swim stress was made of a circular white fiberglass tank (diameter: 100 cm, height: 36 cm) filled with water (temperature: 18 °C, depth: 20 cm). The water tank was placed in a brightly lit testing room. Each animal underwent two sessions of forced swimming, each lasting 1 min. The two swimming sessions were separated by a 3-min interval, during which the animals were kept in a waiting box containing sawdust embedding. The animals were dried with a towel and immediately brought back to their home cages after the second swimming session.

• *Exposure to repeated changing of home cages (day 9).* The last stressor was in the form of repeated changing of home cages. For this purpose, the animals were transferred from their original home cages to new cages containing fresh sawdust embedding. Food and water was supplied to the animals immediately after transferring them from one cage to another in order to avoid additional food or water deprivation. This procedure was repeated 5 times at irregular intervals during the dark phase of the light-dark cycle.

**Behavioral analyses**

• ***Elevated plus maze.*** Innate anxiety-like behavior was assessed using the elevated plus maze test. The apparatus was made of Plexiglas painted in grey and consisted of 4 equally spaced arms (5 x 30 cm^2^) radiating from a square center (5 × 5 cm^2^). One pair of opposing arms was enclosed with opaque walls (height: 15 cm) except for the side adjoining the central square (CZ). The remaining two arms were exposed with a parameter border (height: 3 mm) along the outer edges. The maze was elevated 70 cm above floor level and positioned in a testing room with diffused lighting (approximately 20 Lux in open arm and 10 Lux in closed arm). A digital camera was mounted above the plus maze, captured images at a rate of 5 Hz and transmitted them to a PC running the EthoVision (Noldus Technology, Wageningen, The Netherlands) tracking system. A test session began by placing the animal into the CZ with it facing one of the closed arms. It was then left to explore freely for 5 min before being returned to the home cage. After each trial, the apparatus was cleansed with water and dried before a new trial began. The relative (per cent) open arm entries during the entire 5-min test period were analysed in order to index anxiety-related behaviour. The per cent open arm frequency was calculated using the formula [(open arm entries) / (total arm entries) × 100]. In addition, total distance moved in the entire maze was analysed in order to assess general locomotor activity.

• ***Prepulse inhibition of the acoustic startle reflex.*** Sensorimotor gating was assessed using the paradigm of prepulse inhibition (PPI) of the acoustic startle reflex. PPI of the acoustic startle reflex refers to the reduction in startle reaction in response to a startle-eliciting pulse stimulus when it is shortly preceded by a weak prepulse stimulus. The apparatus consisted of four startle chambers for mice (San Diego Instruments, San Diego, CA, USA) and has been fully described elsewhere (1). During a 45-min test session, subjects were presented with a series of discrete test trials comprising a mixture of four trial-types as fully described previously (1). In brief, these trial stimuli included pulse-alone, prepulse-plus-pulse and prepulse-alone trials, as well as no-stimulus trials in which no discrete stimulus other than the constant background noise (65 dB_A_) was presented. The startle program consisted of one 40-ms pulse of white noise (120 dB_A_) combined with five different pre-pulses. Pre-pulses were a 20-ms burst of white noise at five different intensities (69, 73, 77, 81, and 85 dB_A_, which corresponded to 4, 8, 12, 16, and 20 dB_A_ above background). The stimulus onset asynchrony (SOA) of the pre-pulse and pulse stimuli in prepulse-plus-pulse trials was 100 ms. Following a 2-min acclimatization period, 6 consecutive pulse-alone trials were presented in order to habituate and stabilize the animals' startle response. Subsequent to this startle habituation phase, each trial stimulus was presented 12 times in a pseudorandom order with an average interval between successive trials (ITI) of 15 ± 5 s. The session was concluded with 6 consecutive pulse-alone trials. Boxes were cleansed with warm tap water and dried between each animal. Each box was used by one sex only. For each subject and at each of the five possible prepulse intensities, PPI was indexed by percent inhibition of startle response obtained in the prepulse-plus-pulse-trials compared to pulse-alone trials by following expression: [1-(mean reactivity on prepulse-plus-pulse trials / mean reactivity on pulse-alone trials) × 1/100]. The first and last six trials were not included in the calculation and analysis of percent PPI. In addition to PPI, reactivity to pulse-alone trials and prepulse-alone trials were also analyzed.

• ***Behavioral sensitivity to psychotomimetic drugs.*** The sensitivity to psychotomimetic drugs was assessed by measuring drug-induced locomotor activity in an open field apparatus. The apparatus consisted of 4 identical square arenas (40 × 40 × 35 cm high) made of wood and painted grey as fully described elsewhere (4). First, the animals were intraperitoneally (i.p.) injected with vehicle (isotonic 0.9% NaCl) solution and immediately placed in the apparatus to measure basal locomotor activity for 30 min. Subsequently, the animals were removed from the apparatus and administered with either amphetamine (AMPH; D-amphetamine sulfate, Sigma-Aldrich, Switzerland; 2.5 mg/kg, i.p.) or dizocilpine (MK-801; (+)-MK-801 hydrogen maleate; Sigma-Aldrich, Switzerland; 0.15 mg/kg, i.p.). They were then immediately returned to the same arena again, and the locomotor response to the acute drug challenge was monitored for a period of 90 min. Both drugs were dissolved in isotonic 0.9% NaCl solution to achieve the desired concentration for injection. The doses of AMPH (2.5 mg/kg, i.p.) and MK-801 (0.15 mg/kg, i.p.) were selected based on our previous study showing synergistic effects of the combined prenatal and postnatal manipulations on AMPH- and MK-801-induced changes in locomotor activity (2). The volume of injection was 5 ml/kg for both solutions. All solutions were freshly prepared on the day of testing.

**Immunohistochemical analyses**

The animals were deeply anesthetized with an overdose of Nembutal (Abbott Laboratories) and perfused transcardially with 0.9% NaCl, followed by 4 % phosphate-buffered paraformaldehyde solution containing 15 % picric acid. The dissected brains were postfixed in the same fixative for 6 h and processed for antigen retrieval involving overnight incubation in citric acid buffer (pH = 4.5) followed by a 90 s microwave treatment at 480 W according to protocols established before (2). The brains were then cryoprotected using 30 % sucrose in PBS, frozen with powdered dry ice, and stored at −80 °C until further processing.

Perfused brain samples were cut coronally at 30 μm thickness from frozen blocks with a sliding microtome. Eight serial sections were prepared for each animal and, after rinsing in PBS, stored at −20 °C in antifreeze solution (30% glycerol and 30% ethylene glycol in PBS at 25 mM and pH 7.4) until further processing. For immunohistochemical staining, the slices were rinsed three times for 10 min in PBS, and blocking was done in PBS, 0.3% Triton X-100, 10% normal serum for 1 h at room temperature. The following primary antibodies were used: Rabbit anti-Iba1 (Wako, CatNo# 019-19741; diluted 1:2,000), rat anti-CD68 (AbDSerotec, CatNo# MCA1957; diluted 1:5,000), and rabbit anti-IL-1β (PeproTech, CatNo# 500-P51; diluted 1:1,000). All antibodies were diluted in PBS containing 0.3 % Triton X-100 and 2 % normal serum, and the sections were incubated free-floating overnight at room temperature. After three washes with PBS (10 min each), the sections were incubated for 1 h with the biotinylated secondary antibodies diluted 1:500 in PBS containing 2 % NGS and 0.3 % Triton X-100. Sections were washed again three times for 10 min in PBS and incubated with Vectastain kit (Vector Laboratories) diluted in PBS for 1 h. After three rinses in 0.1 M Tris-HCl, pH 7.4, the sections were stained with 1.25 % 3,3-diaminobenzidine and 0.08% H_2_O_2_ for 10–15 min, rinsed again four times in PBS, dehydrated, and coverslipped with Eukitt (Kindler). The IL-1β-labeled slices were counterstained with 0.25% Cresyl Violet (Nissl stain) according to standard protocols (5).

**Unbiased stereological estimations**

The numbers of Iba1-, CD68, or IL-1β-immunoreactive cells were determined by unbiased stereological estimations using the optical fractionator method (6). With the aid of the image analysis software Stereo Investigator (version 6.50.1; MicroBrightField), every section of a one-in-eight series was measured, resulting in an average of 6 hippocampal and 3 prefrontal cortex sections per brain sample as previously described (2). The following sampling parameters were used: (1) a fixed counting frame with a width of 60 μm and a length of 60 μm; and (2) a sampling grid size of 200 × 150 μm. The counting frames were placed randomly at the intersections of the grid within the outlined structure of interest by the software. The cells were counted following the unbiased sampling rule using the 40× oil lens [numerical aperture (NA), 1.3] and included in the measurement when they came into focus within the optical dissector (7). All immunohistochemical preparations were quantified in the entire (dorsal and ventral) hippocampus with reference to bregma [-1.3 to -3.45 mm] and included CA1 to CA3 subfields and dentate gyrus (DG) as outlined below:

**
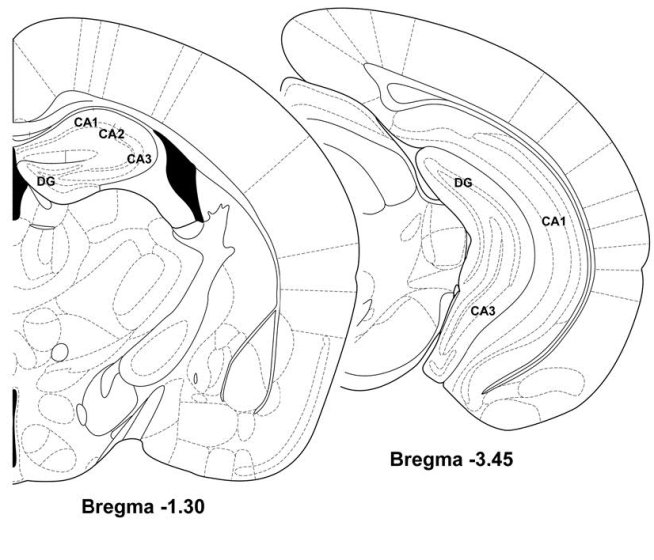
**

Schematic coronal brain sections delineating the hippocampal areas investigated with reference to bregma [adapted from *The Mouse Brain in Stereotaxic Coordinates* (8)].

All immunohistochemical preparations were also quantified in the prefrontal cortex with reference to bregma [+2.3 to +1.7 mm] and included measures from the anterior cingulate (aCG), prelimbic (PrL) and infralimbic (IL) cortices as outlined below:


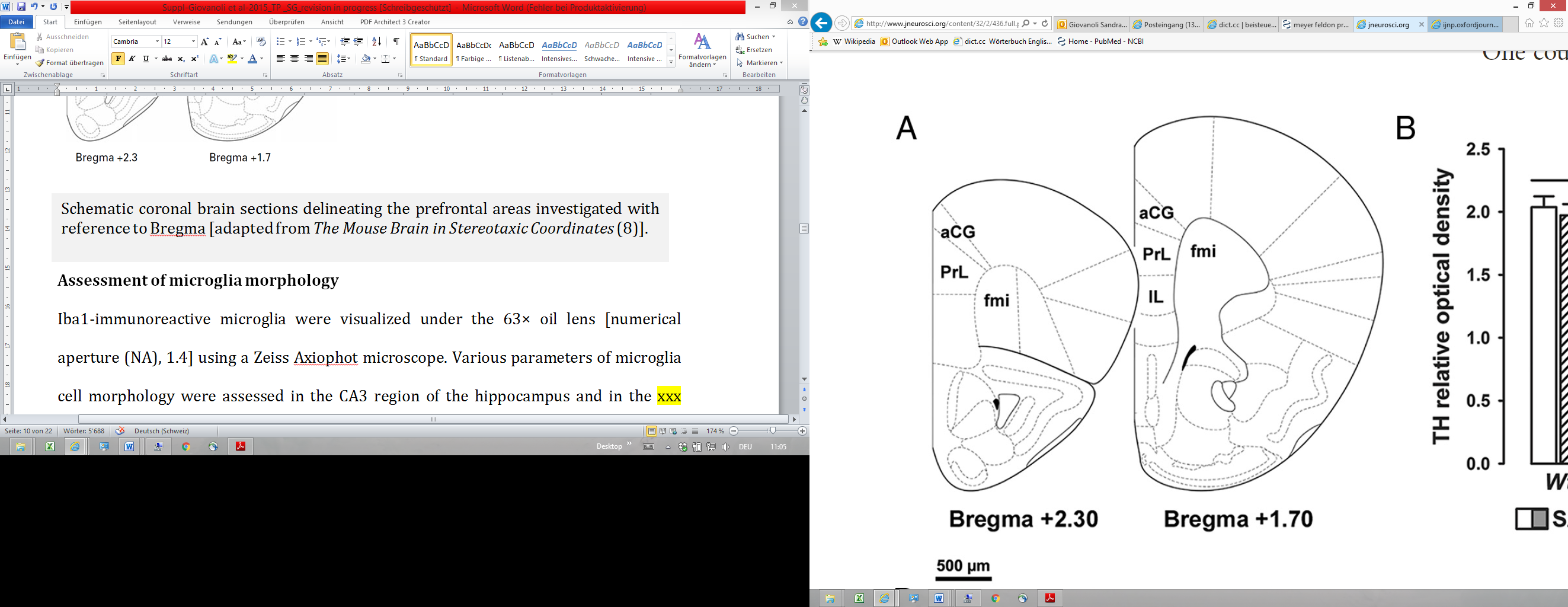


Schematic coronal brain sections delineating the prefrontal areas investigated with reference to bregma [adapted from *The Mouse Brain in Stereotaxic Coordinates* (8)].

**Assessment of microglia morphology**

Iba1-immunoreactive microglia were visualized under the 63× oil lens [numerical aperture (NA), 1.4] using a Zeiss Axiophot microscope. Various parameters of microglia cell morphology were assessed in the CA3 region of the hippocampus and in the prelimbic region of the prefrontal cortex by placing a counting frame of 100 μm x 100 μm randomly into three sections of a one-in-eight series. All microglia cells captured by the counting frame were included in the morphological analyses, except when microglial processes were obscured by either background labeling or other cells. Four microglia cells per section were traced using the software Stereo Investigator (version 6.50.1; MicroBrightField), for which cell soma area and number of primary processes were estimated giving a total of 12 cells per brain region as described before (2).

**Maternal cytokine measurements**

Pregnant mice were killed by decapitation 4 h and 24 h after poly(I:C) or vehicle (saline) administration, and trunk blood was collected into EDTA-treated tubes. The collected blood samples were centrifuged at 2000×g for 5 min and plasma was stored at −20°C until later analyses.

Plasma levels of IL-6 were determined using a bead-based assay (Bio-Plex Pro Mouse Cytokine IL-6 Assay, Bio-Rad Laboratories, Hercules, CA, USA) as previously described (2, 4). Samples were prepared according to the manufacturer's instructions and were analyzed on a triple-laser FACSCanto II flow cytometer using FACSDiva software (BD Immunocytometry Systems, Heidelberg, Germany). Absolute IL-6 concentrations were calculated based on the mean fluorescence intensity of cytokine standards with a 4-parameter logistic curve model using GraphPad Prism 5 (GraphPad Software Inc., La Jolla, CA, USA). The detection limit of the assay was 0.4 pg/ml.

**Preparation of brain samples for pilot gene expression analyses**

To probe the optimal dosing of the minocycline (MINO) treatment with respect to its influence on inflammatory parameters (see main text), we performed initial gene expression analyses evaluating the effects of acute MINO treatment on transcription of neuron-microglia inhibitory signaling pairs and IL-1β (2). Two doses of MINO were included, namely 3 mg/kg or 30 mg/kg (per os, given in regular drinking water across a time span of 24 h), and were compared to vehicle (regular tap water) treatment. The higher dose of MINO (30 mg/kg) was selected based on previous per os dose response studies showing that daily minocycline treatment at this dose and administration regime results in efficient diffusion of the drug across the blood-brain-barrier and accumulation of the drug in the brains of C57BL6 mice (9). The drug was administered on PND 29 for a period of 24 h before the animals were exposed to acute (45-min) restraint stress and immediately sacrificed by decapitation for brain collection. The brains were extracted from the skull and placed ventral side up on an ice-chilled plate for extraction of the left and right hippocampi. The hippocampi were placed in 1-ml Eppendorf tubes, snap frozen in liquid nitrogen, and stored at −80°C until further processing.

**Pilot gene expression analyses of neuron-microglia inhibitory signaling pairs by quantitative real-time PCR**

Total RNA was isolated by a single step guanidinium isothiocyanate/phenol extraction, using PureZol RNA isolation reagent (Bio-Rad Laboratories s.r.l., Italy) according to the manufacturer's instructions, and quantified by spectrophotometric analysis. Following total RNA extraction, the samples were processed for real-time PCR to assess CD200, CD200R, CD47 and CD172a mRNA levels. An aliquot of each sample was treated with DNase to avoid DNA contamination. RNA was analyzed on a qRT–PCR instrument (CFX384 real-time system, Bio-Rad Laboratories) using the iScript one-step RT–PCR kit for probes (Bio-Rad Laboratories). Samples were run in 384-well formats in triplicates as multiplexed reactions with a normalizing internal control (36B4). Probe and primer sequences were purchased from Eurofins MWG-Operon. Thermal cycling was initiated with incubation at 50 °C for 10 min (RNA reverse transcription), and then at 95 °C for 5 min (polymerase activation). After this initial step, 39 cycles of PCR were performed. Each PCR cycle consisted of heating the samples at 95 °C for 10 s to enable the melting process, and then for 30 s at 60 °C for the annealing and extension reactions. Relative target gene expression was calculated according to the 2(-Delta Delta C(T)) method (10).

The following primer and probe sequences were used for the gene expression analysis of neuron-microglia inhibitory signaling pairs:

|  | **FORWARD primer** | **REVERSE primer** | **PROBE** |
| --- | --- | --- | --- |
| CD200 | CTCCACCTACGCCTGATTTG | TCACAATCAAGGGTTCCTGG | AGCACAGCTCAAGTGGAAGTGGT |
| CD200R | TTTTGGAGAACTTCTGCCCTAG | GTGTTCACTTGTGTCAGAGGA | ACTTGACCCAGCCACAAAGACCC |
| CD47 | AGGAGAAAAGCCCGTGAAG | TGGCAATGGTGAAAGAGGTC | ACAATGAGGCCAAGTCCAGAAGCA |
| CD172a | TGTGCTTTGCTCGTAGTCC | TCATTTGTGTCCTGGATCTGG | TGTTGACCCCTTGGCTTTCTTCTGT |
| 36B4 | AGATGCAGCAGATCCGCAT | GTTCTTGCCCATCAGCACC | CGCTCCGAGGGAAGGCCG |

**Pilot gene expression analysis of IL-1β by quantitative real-time PCR**

Brain samples were homogenized with a rotor/stator tissue homogenizer (Tissue-Tearor™, BioSpec Products, Bartlesville, OK, USA) in TRI Reagent® (Sigma-Aldrich), and total RNA was isolated according to the manufacturer’s instructions. Further purification and DNase I treatment was performed using the RNeasy Micro kit (Qiagen, Hilden, Germany). Concentrations were determined photometrically in a BioPhotometer (Eppendorf, Hamburg, Germany) using a LabelGuard Microliter Cell (Implen, Munich, Germany). First-strand cDNA was synthesized from 1 μg of total RNA using the High Capacitiy cDNA Reverse Transcription Kit (Applied Biosystems). Real-time quantitative PCR was performed on a 7500 Fast Real-Time PCR System (Applied Biosystems) using the FAST qPCR MasterMix Plus Low ROX with UNG (Eurogentec, Seraing, Belgium) and the following cycling conditions: 2 min at 50 °C and 5 min at 95 °C followed by 40 cycles of 3 sec at 95 °C and 40 sec at 60 °C. Primers and probe for IL-1β were adapted from the RTPrimerDatabase (<http://medgen.ugent.be/rtprimerdb/>) and were purchased from Eurogentec (Seraing, Belgium). 36B4 was used as reference gene as for above, and relative gene expression was calculated according to the 2(-Delta Delta C(T)) method (10).

The following primer and probe sequences (5’-3’) were used for cytokine gene expression analysis:

|  | **FORWARD primer** | **REVERSE Primer** | **PROBE**  **(5’-6-FAM and 3’-BHQ1)** |
| --- | --- | --- | --- |
| IL-1β | CAACCAACAAGTGATATTCTCCATG | GATCCACACTCTCCAGCTGCA | CTGTGTAATGAAAGACGGCACACCCACC |
| 36B4 | AGATGCAGCAGATCCGCAT | GTTCTTGCCCATCAGCACC | CGCTCCGAGGGAAGGCCG |

**Supplementary Results**


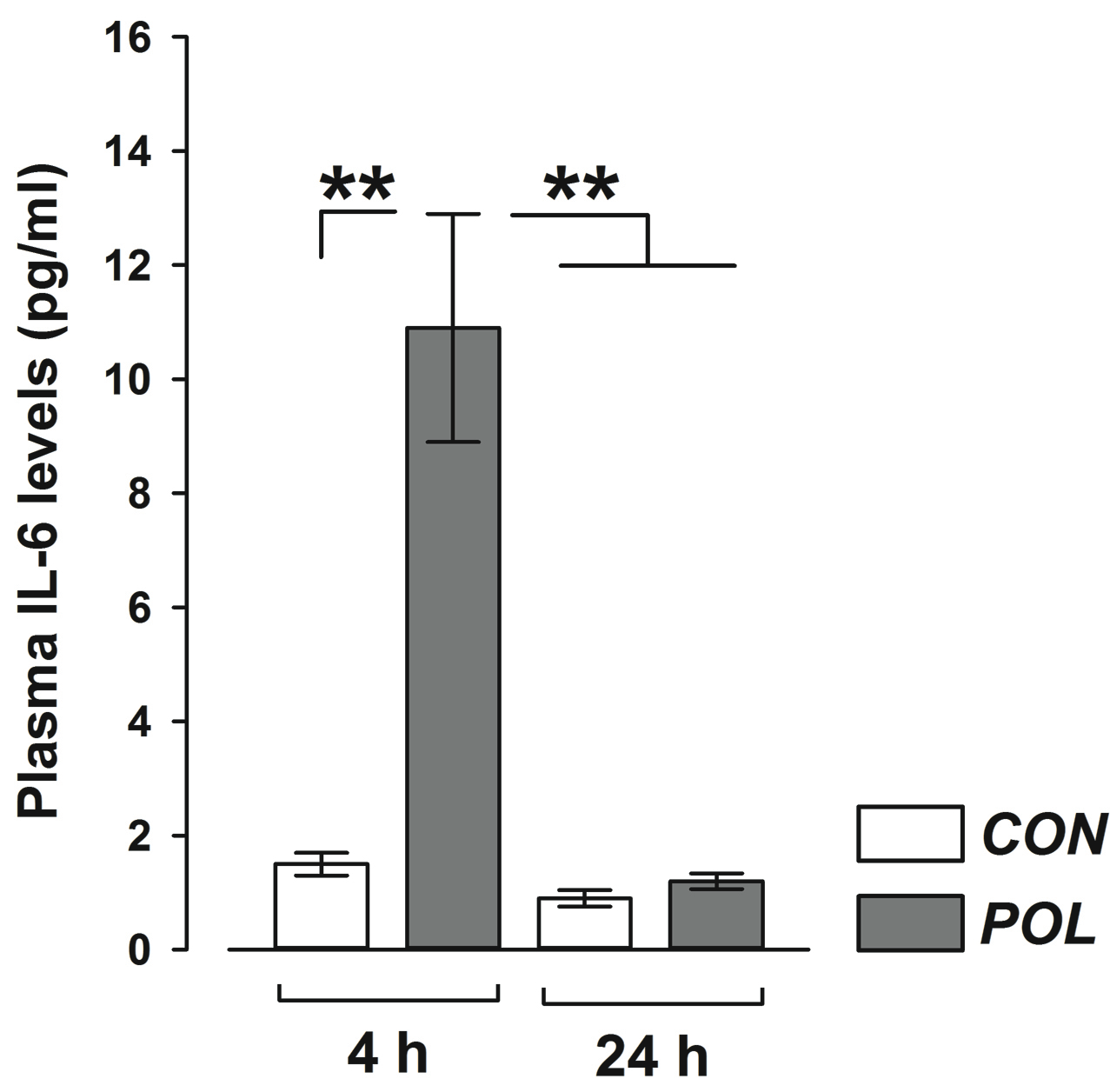


***Supplementary Figure 1.*** Maternal IL-6 levels at basal conditions and following viral-like immune activation in mid-pregnancy. Pregnant mice were exposed to poly(I:C) (1 mg/kg,i.v.; = POL) or control (= CON) vehicle (saline) solution on gestation day 9, and IL-6 plasma levels were measured 4 and 24 h post-treatment. Note that the POL-induced elevation of cytokines was transient, returning to basal levels within 24 h post-treatment. ***P*< 0.01, based on post-hoc comparisons, following presence of a significant interaction between maternal treatment and sampling interval (F_(1,16)_ = 16.27, *P*< 0.01). *N* = 5 animals per treatment group/post-injection interval. All data are means±s.e.m.


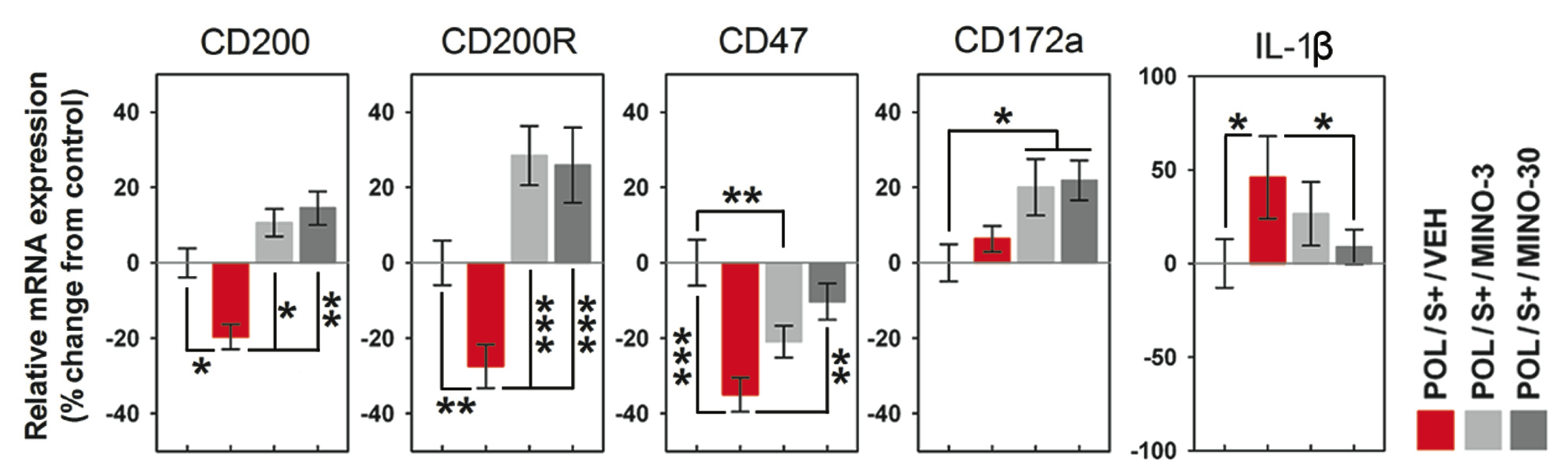


***Supplementary Figure 2****.* Effects of minocycline (MINO) treatment on neuron–microglia inhibitory signaling pairs (CD200, CD200R; CD47, CD172a) and IL-1β gene expression in the hippocampus of offspring exposed to combined prenatal immune activation and peripubertal stress relative to control offspring. We have previously documented that the combination of prenatal immune activation by poly(I:C) (= POL) and peripubertal stress exposure (= S+) markedly impairs the expression of CD200, CD200R, and CD47, and leads to increased expression of IL-1β (see reference [2]). Here, we explored whether MINO treatment can restore these deficits. Therefore, POL/S+ offspring were treated with 3 mg/kg/day minocycline (MINO-3), 30 mg/kg/day minocycline (MINO-30), or vehicle (VEH = regular tap water) for 24 hrs prior to stress exposure, and mRNA levels were compared to VEH-treated CON/S- (= control) animals. **P*< 0.05, ***P*< 0.01, and ****P*< 0.001, based on student’s *t* test (two-tailed) following one-way ANOVA. *N*=8-10 males per group. All data are means±s.e.m.

***
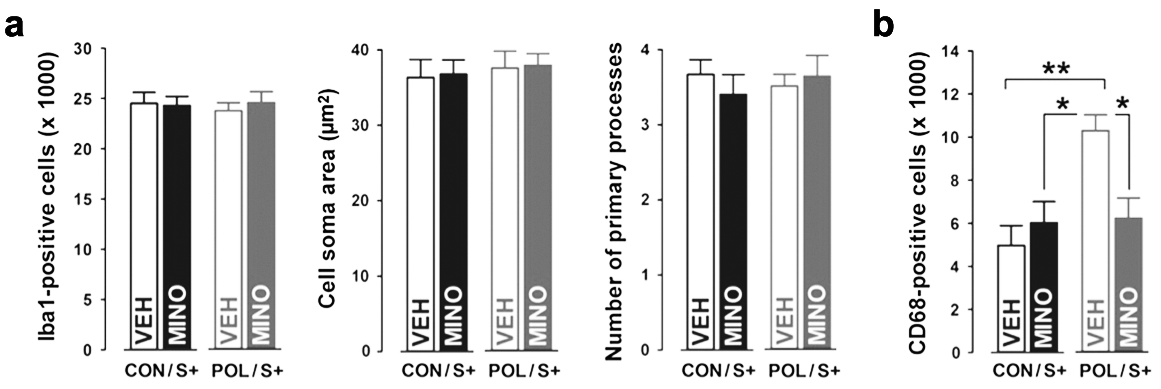
***

***Supplementary Figure 3*.** Effects of minocycline (MINO) on microglia abnormalities in the prefrontal cortex of stressed offspring born to control or gestationally immune challenged mothers. Pregnant mice were exposed to poly(I:C) (POL) or control (CON) solution, and the resulting offspring were subjected to sub-chronic stress (S+) during peripubertal maturation. During the stress procedure, half of the animals received MINO treatment, and the other half vehicle (VEH = regular tap water) treatment. **(a)** The bar plots depict the stereological estimates of Iba1-positive cells as well as cell soma area and number of primary processes of Iba1-positive microglia. *N* = 5 per group. **(b)** The bar plot shows the stereological estimates of activated CD68-positive microglia cells. **P* < 0.05, ***P* < 0.01; *N* = 5 per group. All data are means±s.e.m.

| **Markers** | **CON/S-/**  **VEH** | **POL/S+/**  **VEH** | **POL/S+/**  **MINO 30** | **POL/S+/**  **MINO 30** |
| --- | --- | --- | --- | --- |
| ***CD200,  CD200R CD47 CD172a***  ***IL-1beta*** | 10m | 8m | 8m | 8m |

***Supplementary Table 1.*** Summary of the number of offspring used for the pilot gene expression experiments. Offspring born to polyI:C treated mothers (POL) were treated with 3 mg/kg minocycline (MINO-3), 30 mg/kg minocycline (MINO-30), or vehicle (VEH = regular tap water) for 24 hrs prior to stress exposure (S+), and mRNA levels were compared to VEH-treated CON/S- (= control) animals. Only male (m) subjects were used in this experiment.

| **Test** | **CON/S-/**  **VEH** | **CON/S-/**  **MINO** | **POL/S-/**  **VEH** | **POL/S-**  **MINO** | **CON/S+/**  **VEH** | **CON/S+/**  **MINO** | **POL/S+/**  **VEH** | **POL/S+/**  **MINO** |
| --- | --- | --- | --- | --- | --- | --- | --- | --- |
| **EPM PPI** | **15** (7f, 8m) | **12** (6f, 6m) | **18**  (10f, 8m) | **16** (9f, 7m) | **16**  (7f, 9m) | **15** (7f, 8m) | **15** (8f, 7m) | **12** (6f, 6m) |
| **AMPH** |  |  |  |  | **16** (8f, 8m) | **15** (7f, 8m) | **14** (7f, 7m) | **12** (7f, 5m) |
| **MK-801** |  |  |  |  | **16**  (8f, 8m) | **16**  (8f, 8m) | **16**  (8f, 8m) | **16**  (8f, 8m) |

***Supplementary Table 2.*** Summary of the number of female (f) and male (m) offspring used in the behavioral experiments. Pregnant mice were exposed to poly(I:C) (POL) or control (CON) solution, and the resulting offspring were subjected to sub-chronic stress (S+) or left undisturbed (S-) during peripubertal development. During the stress procedure, half of the animals received minocycline (MINO) treatment, and the other half vehicle (VEH = regular tap water) treatment. The AMPH and MK-801 tests were conducted using the stress condition only.

| **Test** | **Dependent Measures** | **ANOVA** | **Effects** | **DF** | **F-Value** | **P-Value** |
| --- | --- | --- | --- | --- | --- | --- |
| **Elevated plus maze** | Relative open arm entries (%) | 2 × 2 × 2 × 2  (Sex × MIA × Stress × Prevention) | Sex | (1, 103) | 1.211 | 0.2737 |
|  |  |  | MIA | (1, 103) | 3.125 | 0.0801 |
|  |  |  | Stress | (1, 103) | 14.387 | **0.0003** |
|  |  |  | Prevention | (1, 103) | 2.559 | 0.1127 |
|  |  |  | MIA × Stress × Prevention | (1, 103) | 0.377 | 0.5403 |
|  |  |  | Sex × MIA × Stress × Prevention | (1, 103) | 0.733 | 0.3938 |
|  | Total distance moved (cm) | 2 × 2 × 2 × 2  (Sex × MIA × Stress × Prevention) | Sex | (1, 103) | 1.940 | 0.1667 |
|  |  |  | MIA | (1, 103) | 3.063 | 0.0831 |
|  |  |  | Stress | (1, 103) | 1.112 | 0.2942 |
|  |  |  | Prevention | (1, 103) | 0.035 | 0.8522 |
|  |  |  | MIA × Stress × Prevention | (1, 103) | 1.063 | 0.3051 |
|  |  |  | Sex × MIA × Stress × Prevention | (1, 103) | 0.166 | 0.6848 |
| **Prepulse inhibition** | Percent PPI (%) | 2 × 2 × 2 × 2 × 5  (Sex × MIA × Stress × Prevention × Prepulse) | Sex | (1, 103) | 0.092 | 0.7622 |
|  |  |  | MIA | (1, 103) | 7.474 | **0.0074** |
|  |  |  | Stress | (1, 103) | 3.734 | 0.0561 |
|  |  |  | Prevention | (1, 103) | 4.036 | **0.0472** |
|  |  |  | MIA × Stress × Prevention | (1, 103) | 12.285 | **0.0007** |
|  |  |  | Sex × MIA × Stress × Prevention | (1, 103) | 0.014 | 0.9070 |
|  |  |  | Prepulse | (4, 412) | 273.681 | <**0.0001** |
|  | Startle reactivity (AU) | 2 × 2 × 2 × 2  (Sex × MIA × Stress × Prevention) | Sex | (1, 103) | 21.176 | <**0.0001** |
|  |  |  | MIA | (1, 103) | 3.803 | 0.0539 |
|  |  |  | Stress | (1, 103) | 1.307 | 0.2557 |
|  |  |  | Prevention | (1, 103) | 0.146 | 0.7034 |
|  |  |  | MIA × Stress × Prevention | (1, 103) | 1.605 | 0.2081 |
|  |  |  | Sex × MIA × Stress × Prevention | (1, 103) | 1.479 | 0.2267 |
|  | Prepulse induced reactivity (AU) | 2 × 2 × 2 × 2 × 5  (Sex × MIA × Stress × Prevention × Prepulse) | Sex | (1, 103) | 2.771 | 0.0990 |
|  |  |  | MIA | (1, 103) | 1.015 | 0.3160 |
|  |  |  | Stress | (1, 103) | 1.011 | 0.3169 |
|  |  |  | Prevention | (1, 103) | 2.538 | 0.1142 |
|  |  |  | MIA × Stress × Prevention | (1, 103) | 2.689 | 0.1041 |
|  |  |  | Sex × MIA × Stress × Prevention | (1, 103) | 2.099 | 0.1504 |
|  |  |  | Prepulse | (4, 412) | 79.332 | <**0.0001** |
| **Locomotor response to AMPH** | Total distance moved following vehicle (cm) | 2 × 2 × 2 × 6  (Sex × Group × Prevention × Bins) | Sex | (1, 49) | 2.002 | 0.1634 |
|  |  |  | Group | (1, 49) | 0.386 | 0.5372 |
|  |  |  | Prevention | (1, 49) | 0.588 | 0.4469 |
|  |  |  | Group × Prevention | (1, 49) | 0.334 | 0.5662 |
|  |  |  | Sex × Group × Prevention | (1, 49) | 0.240 | 0.6262 |
|  |  |  | Bins | (5,245) | 37.333 | <**0.0001** |
|  |  |  | Bins × Group × Prevention | (5,245) | 0.523 | 0.7589 |
|  |  |  | Bins × Sex × Group × Prevention | (5,245) | 0.780 | 0.5650 |
|  | Total distance moved following AMPH (cm) | 2 × 2 × 2 × 18  (Sex × Group × Prevention × Bins) | Sex | (1, 49) | 2.261 | 0.1391 |
|  |  |  | Group | (1, 49) | 9.415 | **0.0035** |
|  |  |  | Prevention | (1, 49) | 2.679 | 0.1081 |
|  |  |  | Group × Prevention | (1, 49) | 12.013 | **0.0011** |
|  |  |  | Sex × Group × Prevention | (1, 49) | 2.545 | 0.1170 |
|  |  |  | Bins | (17,833) | 38.305 | <**0.0001** |
|  |  |  | Bins × Group × Prevention | (17,833) | 6.765 | <**0.0001** |
|  |  |  | Bins × Sex × Group × Prevention | (17,833) | 0.640 | 0.8614 |
| **Locomotor response to MK** | Total distance moved following vehicle (cm) | 2 × 2 × 2 × 6  (Sex × Group × Prevention × Bins) | Sex | (1,56) | 2.637 | 0.1100 |
|  |  |  | Group | (1,56) | 0.013 | 0.9085 |
|  |  |  | Prevention | (1,56) | 1.276 | 0.2634 |
|  |  |  | Group × Prevention | (1,56) | 0.001 | 0.9749 |
|  |  |  | Sex × Group × Prevention | (1,56) | 1.523 | 0.2223 |
|  |  |  | Bins | (5,280) | 69.219 | <**0.0001** |
|  |  |  | Bins × Group × Prevention | (5,280) | 0.765 | 0.5755 |
|  |  |  | Bins × Sex × Group × Prevention | (5,280) | 0.153 | 0.9791 |
|  | Total distance moved following MK-801 (cm) | 2 × 2 × 2 × 18  (Sex × Group × Prevention × Bins) | Sex | (1,56) | 2.794 | 0.1002 |
|  |  |  | Group | (1,56) | 1.319 | 0.2556 |
|  |  |  | Prevention | (1,56) | 1.909 | 0.1726 |
|  |  |  | Group × Prevention | (1,56) | 0.525 | 0.4716 |
|  |  |  | Sex × Group × Prevention | (1,56) | 0.066 | 0.7985 |
|  |  |  | Bins | (17,952) | 15.334 | <**0.0001** |
|  |  |  | Bins × Group × Prevention | (17,952) | 1.648 | **0.0470** |
|  |  |  | Bins × Sex × Group × Prevention | (17,952) | 0.871 | 0.6077 |

***Supplementary Table 3 (previous page).*** Summary of the statistical tests and outcomes for the behavioral analyses. The table specifies the dependent measures for each behavioral test and summarizes the main effects of and interactions between the main between-subjects factors (Sex; maternal immune activation = MIA; peripubertal stress = Stress; minocycline treatment = Prevention), as well as the main effects of and interactions between additional independent factors specific to each behavioral test. Note that the AMPH and MK-801 tests were conducted using the stress condition only, so that the MIA/stress condition appears as “Group”. The table also specifies the corresponding degrees of freedom (DF) and F-values. Significant effects (*P*< 0.05) are given in bold font. AU, arbitrary units; AMPH, amphetamine; MK-801, dizocilpine; PPI, prepulse inhibition.

| **Cellular Marker** | **Dependent Measures** | **Effects** | **DF** | **F-Value** | **P-Value** |
| --- | --- | --- | --- | --- | --- |
| **Iba1** | Number of cells  (cells/mm^3^) | Group | (1,16) | 0.223 | 0.6429 |
|  |  | Prevention | (1,16) | 1.598 | 0.2243 |
|  |  | Group × Prevention | (1,16) | 1.674 | 0.2141 |
| **Iba1 Cell morphology** | Cell soma area (μm^2^) | Group | (1,16) | 0.002 | 0.9676 |
|  |  | Prevention | (1,16) | 0.202 | 0.6590 |
|  |  | Group × Prevention | (1,16) | 4.516 | **0.0495** |
|  | Number of primary processes  (primary process/cell) | Group | (1,16) | 0.095 | 0.7617 |
|  |  | Prevention | (1,16) | 0.522 | 0.4805 |
|  |  | Group × Prevention | (1,16) | 4, E^-4^ | 0.9842 |
| **CD68** | Number of cells  (cells/mm^3^) | Group | (1,16) | 0.193 | 0.6660 |
|  |  | Prevention | (1,16) | 1.025 | 0.3264 |
|  |  | Group × Prevention | (1,16) | 7.709 | **0.0135** |
| **IL-1β** | Number of cells  (cells/mm^3^) | Group | (1,16) | 0.352 | 0.5611 |
|  |  | Prevention | (1,16) | 0.900 | 0.3569 |
|  |  | Group × Prevention | (1,16) | 5.918 | **0.0271** |

***Supplementary Table 4.*** Summary of the statistical tests and outcomes for the hippocampal immunohistochemcial analyses conducted in peripubertal (postnatal day 41) offspring following combined prenatal immune activation and stress with or without preventive minocycline treatment. The table summarizes the main effects of and interactions between the main between-subjects factors (combined maternal immune activation and stress = Group; preventive minocycline administration = Prevention). The table also specifies the corresponding degrees of freedom (DF) and F-values. Only male subjects were included in the immunohistochemcial analyses. Significant effects (*P*< 0.05) are given in bold font.

| **Cellular Marker** | **Dependent Measures** | **Effects** | **DF** | **F-Value** | **P-Value** |
| --- | --- | --- | --- | --- | --- |
| **Iba1** | Number of cells  (cells/mm^3^) | Group | (1,16) | 0.068 | 0.7970 |
|  |  | Prevention | (1,16) | 0.260 | 0.6171 |
|  |  | Group × Prevention | (1,16) | 0.073 | 0.7899 |
| **Iba1 Cell morphology** | Cell soma area (μm^2^) | Group | (1,16) | 0.967 | 0.3400 |
|  |  | Prevention | (1,16) | 0.003 | 0.9591 |
|  |  | Group × Prevention | (1,16) | 0.102 | 0.7537 |
|  | Number of primary processes  (primary process/cell) | Group | (1,16) | 0.039 | 0.8468 |
|  |  | Prevention | (1,16) | 0.154 | 0.7002 |
|  |  | Group × Prevention | (1,16) | 1.321 | 0.2673 |
| **CD68** | Number of cells  (cells/mm^3^) | Group | (1,16) | 3.709 | 0.0721 |
|  |  | Prevention | (1,16) | .0507 | 0.4868 |
|  |  | Group × Prevention | (1,16) | 10.520 | **0.0051** |

***Supplementary Table 5.*** Summary of the statistical tests and outcomes for the prefrontal immunohistochemcial analyses conducted in peripubertal (postnatal day 41) offspring following combined prenatal immune activation and stress with or without preventive minocycline treatment. The table summarizes the main effects of and interactions between the main between-subjects factors (combined maternal immune activation and stress = Group; preventive minocycline administration = Prevention). The table also specifies the corresponding degrees of freedom (DF) and F-values. Only male subjects were included in the immunohistochemcial analyses. Significant effects (*P*< 0.05) are given in bold font.

| **Group** | **Mean Startle Reaction to the Pulse (AU) ± S.E.M.** | **Mean Startle Reaction to the Prepulses (AU) ± S.E.M** |
| --- | --- | --- |
| **SAL/S-/VEH** | 94.1 ± 11.5 | 3.7 ± 0.33 |
| **SAL/S+/VEH** | 89.8 ± 10.3 | 5.9 ± 0.96 |
| **SAL/S-/MINO** | 76.5 ± 16.4 | 4.9 ± 0.84 |
| **SAL/S+/MINO** | 101.9 ± 11.2 | 4.7 ± 0.98 |
| **POL/S-/VEH** | 91.2 ± 10.1 | 5.0 ± 0.44 |
| **POL/S+/VEH** | 106.4 ± 13.8 | 4.8 ± 0.50 |
| **POL/S-/MINO** | 106.8 ± 11.8 | 3.7 ± 0.39 |
| **POL/S+/MINO** | 110.6 ± 15.5 | 3.7 ± 0.44 |

***Supplementary Table 6.*** Summary of the mean±S.E.M startle reactivity (= reaction to pulse alone trials) and prepulse-induced reactivity (= reaction to prepulse-alone trials). Pregnant mice were exposed to poly(I:C) (POL) or control (CON) solution, and the resulting offspring were subjected to sub-chronic stress (S+) or left undisturbed (S-) during peripubertal development. During the stress procedure, half of the animals received minocycline (MINO) treatment, and the other half vehicle (VEH = regular tap water) treatment. No significant group differences were obtained for these measures. *N*=12-18 per group. AU, arbitrary units.

**REFERENCES**

1. Meyer U, Feldon J, Schedlowski M, Yee BK. Towards an immuno-precipitated neurodevelopmental animal model of schizophrenia. *NeurosciBiobehav Rev* 2005; **29:** 913-913-47.
2. Giovanoli S, Engler H, Engler A, Richetto J, Voget M, Willi R, Winter C, Riva MA, Mortensen PB, Feldon J, Schedlowski M, Meyer U. Stress in puberty unmasks latent neuropathological consequences of prenatal immune activation in mice. *Science* 2013; **339:** 1095–9.
3. Clancy B, Finlay BL, Darlington RB, Anand KJ. Extrapolating brain development from experimental species to humans. *Neurotoxicology* 2007; **28:** 931-7.
4. Meyer U, Murray PJ, Urwyler A, Yee BK, Schedlowski M, Feldon J. Adult behavioral and pharmacological dysfunctions following disruption of the fetal brain balance between pro-inflammatory and IL-10-mediated anti-inflammatory signaling. *Mol Psychiatry* 2008; **13:** 208-21.
5. Nyffeler M, Meyer U, Yee BK, Feldon J, Knuesel I. Maternal immune activation during pregnancy increases limbic GABAA receptor immunoreactivity in the adult offspring: implications for schizophrenia. *Neuroscience* 2006; **143:** 51-62.
6. Gundersen HJ, Bagger P, Bendtsen TF, Evans SM, Korbo L, Marcussen N, Møller A, Nielsen K, Nyengaard JR, Pakkenberg B *et al*. The new stereological tools: disector, fractionator, nucleator and point sampled intercepts and their use in pathological research and diagnosis. *APMIS* 1988; **96:** 857-81.
7. Howard CV, Reed MG. Garland Science/BIOS Scientific, New York 2005.
8. Franklin KBJ, Paxinos G. *The Mouse Brain in Stereotaxic Coordinates.* Academic Press: San Diego, CA 2008.
9. Lee CZ, Yao JS, Huang Y, Zhai W, Liu W, Guglielmo BJ, Lin E, Yang GY, Young WL. Dose-response effect of tetracyclines on cerebral matrix metalloproteinase-9 after vascular endothelial growth factor hyperstimulation. *J Cereb Blood Flow Metab* 2006; **26:** 1157–64.
10. Livak KJ, Schmittgen TD. Analysis of relative gene expression data using real-time quantitative PCR and the 2(-Delta Delta C(T)) Method. *Methods* 2001; **25:** 402-8.
